# Supplementary material for: Thinning Antarctic glaciers expose high-altitude nunataks delivering more bioavailable iron to the Southern Ocean
Source: Nat Commun. 2025 Nov 24;16:9994. doi: 10.1038/s41467-025-65714-y (PMC12645001; doi:10.1038/s41467-025-65714-y)
Supplement: Supplementary file 1 — Supplementary Information [file 41467_2025_65714_MOESM1_ESM.pdf]

Supplementary Information:

Thinning Antarctic glaciers expose high-altitude nunataks delivering more bioavailable iron to the Southern Ocean

Kate Winter, John Woodward, Stuart A. Dunning, James R. Jordan, Joseph A. Graly, Matthew J. Westoby, Sian F. Henley, Robert Raiswell

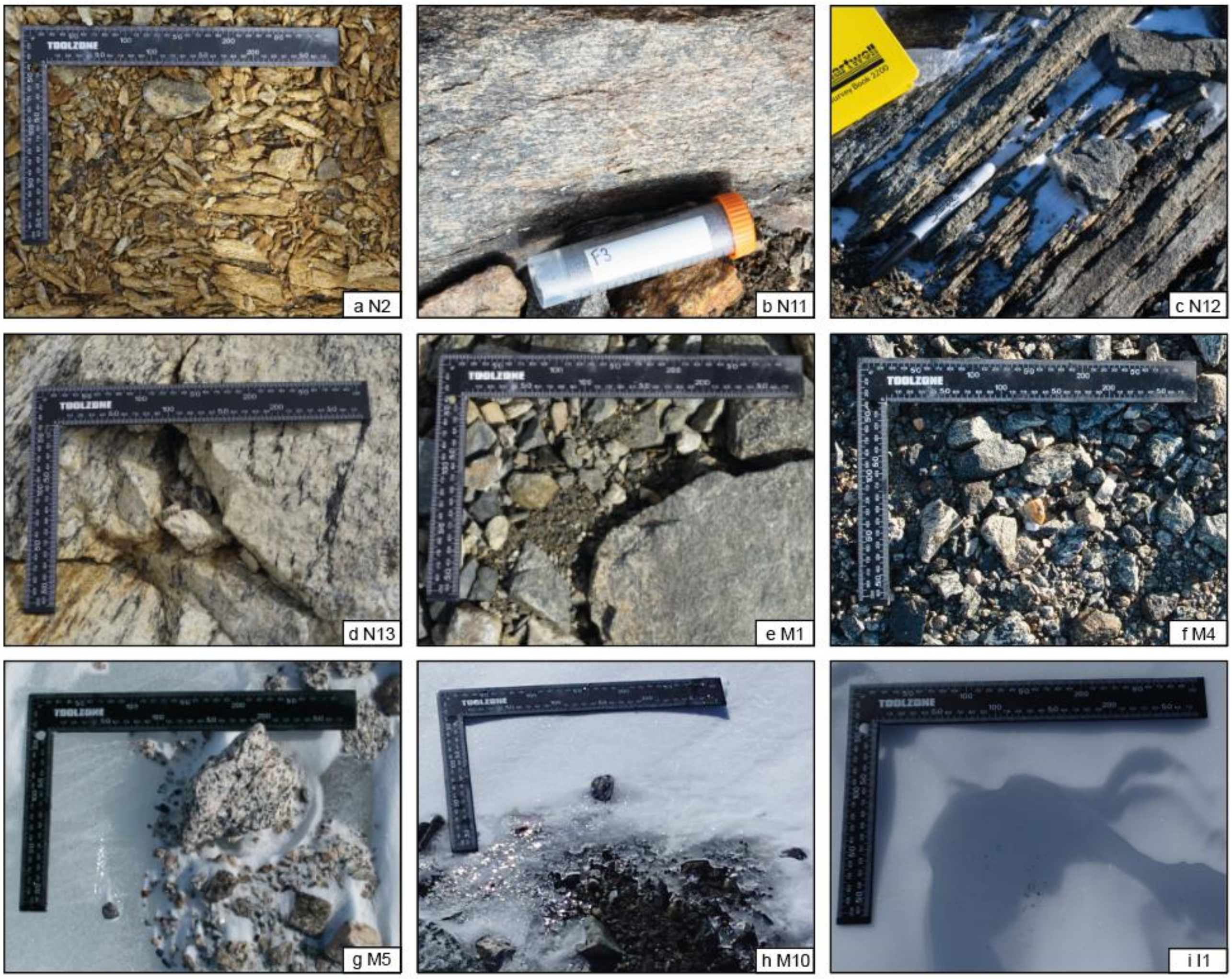

Supplementary Figure 1. Photographs of Antarctic sediments sampled from nunataks (a-d), moraines (e-h), including blue ice moraines, and the ice surface (i). Sample locations are shown in Figure 1c, and sample geochemistry is detailed in Table 1. Supplementary Data provide all sample locations and geochemistry data. The black engineer's square measures 30 cm across and 20 cm down. The sample bottle placed in b is ~12 cm long and the pen in c is ~14 cm long, for scale.

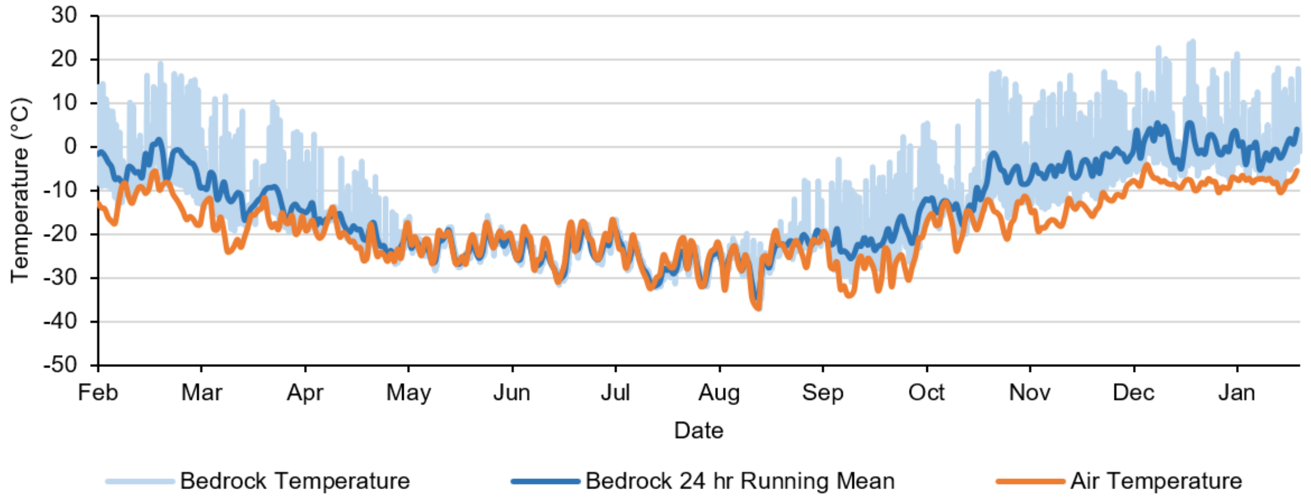

Supplementary Figure 2. Temperature records from Dronning Maud Land, East Antarctica from 1st February 2019 – 20th January 2020. Hourly bedrock temperature (light blue) was measured using a MadgeTech CryoTemp Ultra Low Temperature Data Logger installed onto a nunatak spur located at 72.0891°S, 23.4397°E (next to sediment sample N11, Figure 1c), where the dark blue line marks the 24-hour running mean bedrock temperature. Air temperature was measured from a local automatic weather station called "PEA_AIR_AWS" installed by Konrad Steffen (Swiss Federal Institute for Forest, Snow and Landscape Research) at 71.9474°S, 23.3009°E, near Utseinen Nunatak (marked in Figure 1), where data are presented as daily mean values (orange line).
