# Supplementary material for: Thinning Antarctic glaciers expose high-altitude nunataks delivering more bioavailable iron to the Southern Ocean
Source: Nat Commun. 2025 Nov 24;16:9994. doi: 10.1038/s41467-025-65714-y (PMC12645001; doi:10.1038/s41467-025-65714-y)
Supplement: Supplementary file 3 — Description of Additional Supplementary Files [file 41467_2025_65714_MOESM3_ESM.pdf]

Description of Additional Supplementary Files

File Name: Supplementary Data 1
Description: Geochemical data generated in this study

File Name: Supplementary Data 2
Description: Rock/air temperature data described in the paper and supplementary information file.
